# Synthesis of water soluble ionic liquid copolymers based on acrylamide and investigation of their properties in flocculating of clay suspensions

**DOI:** 10.1038/s41598-023-41547-x

**Published:** 2023-08-30

**Authors:** Shirin Faridi, Akbar Mobinikhaledi, Hassan Moghanian, Meisam Shabanian

**Affiliations:** 1 Department of Chemistry, Faculty of Science, Arak University, Arak, 38156-8-8349 Iran; 2 Materials and Energy Research Center, Dezful Branch, Islamic Azad University, Dezful, Iran; 3 Faculty of Chemistry and Petrochemical Engineering, Standard Research Institute (SRI), P.O. Box 31745-139, Karaj, Iran

**Keywords:** Chemistry, Materials science

## Abstract

To overcome water absorption and swelling by clay mineral layers, it is very important to develop stabilizing additives for water-based drilling fluids, where organic polymers are used as raw materials. Acrylamide copolymers, acting as flocculating agents, have the potential to separate minerals such as montmorillonite. In this study, three water-soluble copolymers containing acrylamide-amphoter, acrylamide-amphoter-anion, and acrylamide-amphoter-cation were synthesized and characterized using various analytical techniques, including Fourier-transform infrared spectroscopy, nuclear magnetic resonance, gel permeation chromatography, differential scanning calorimetry, thermogravimetric analysis, and derivative thermal gravimetric techniques. These copolymers were employed as flocculants to treat water suspensions containing montmorillonite particles, and a range of analytical methods, such as sedimentation volume measurement, scanning electron microscopy analysis, X-ray diffraction analysis, and contact angle measurement, were employed to identify the relationship between inhibitive performance. The flocculation of montmorillonite plates was attributed to the electrostatic attractions between montmorillonite and the synthesized copolymers. High molecular weight copolymers offer greater thermal stability and better flocculation characteristics for water-based drilling fluids. Among the tested copolymers, the acrylamide-amphoter-anion sample, with the highest molecular weight, exhibited the best performance as a coagulant when compared to the other copolymers.

## Introduction

Drilling muds consist of suspensions of montmorillonite particles in water or oil which is an inseparable part of the drilling process. Most drilling muds, which are currently used in oil fields, formulated in water^1–3^. Shale hydration during drilling using water-based drilling fluids is a major problem^4^. Flocculants are essential to minimize swelling of the montmorillonite shale during drilling^5^. The synthetic flocculants are more effective than the natural flocculants due to their efficiency and low cost^6^. Research has indicated that the efficiency and nature of flocculation are influenced by various factors such as the structure, molecular weight, charge, and dosage of the flocculant. For example, in systems where bridging is the primary mechanism of flocculation, the flocculation process can be significantly enhanced by using a high molecular weight polyelectrolyte, regardless of its charge^7–9^. In addition to bridging mechanisms, flocculation can also occur through charge neutralization, specifically through the patch mechanism. In such cases, a low molecular weight polyelectrolyte is typically used. When using a cationic polyelectrolyte in the presence of negatively charged clays, the main driving force is electrostatic attraction^10^. Several materials have been investigated as potential shale inhibitors but most of them suffer degradation at drilling which hinders their potential applications^11^. Flocculants generally perform well within specific temperature ranges. Also, the pH of the environment can significantly impact the performance of flocculants. Some polymer flocculants exhibit notable sensitivity to the salinity of the fluid^12,13^. Various water soluble polymers have been used in the water-based drilling fluid to prevent fluid loss. The high temperature causes the failure of drilling fluid treatment agent^14^. Therefore, it is necessary to design heat-resistant and ecofriendly flocculants^15^. Acrylamide copolymers are used as drilling fluid additives to overcome wall instability and collapse problems^16^. Many research works have indicated that acrylamide copolymerized with a suitable monomer has high thermal stability^17^. Most flocculants are acrylamide-based polymers and may be available in anionic, cationic, nonionic, or amphoteric forms^18^. The copolymer adsorption on the surface of montmorillonite particles generally depends on the functional group of monomers and charge distribution^19–21^. The interaction between acrylamide copolymers and montmorillonite sheets occurs through electrostatic attractions. Therefore, as the type of charged functional groups in the acrylamide copolymer chain changes, their interactions with montmorillonite also changes^22–24^. Also, the main problem of the current world is the lack of fresh water, which is due to the increase in environmental pollution. Flocculation is an effective and economic method for wastewater treatment. There are many flocculants of treating wastewater, which are used in order to remove organic and inorganic materials^15,25,26^. Neglecting the treatment of these dispersions can have detrimental effects, not only resulting in the wastage of valuable water resources but also causing significant environmental issues^27^. Polyacrylamide and its copolymers are extensively employed in water treatment and sludge dewatering. The efficiency of flocculation is influenced by factors such as the type of flocculant used, its molecular weight, and its concentration in the water suspension. Flocculation occurs through mechanisms of charge neutralization and bridging. The flocculation process is considered effective and satisfactory when there is a noticeable decrease in turbidity and accelerated settling of particles^28–31^. In the present research, copolymers containing AA, DMAPS, AMPS and DADMAC monomers have been prepared and applied as coagulant additives in water-based drilling fluids. These copolymers can largely prevent the hydration swelling of montmorillonite minerals, and on the other hand, they cause coagulation by creating electrostatic attraction forces with montmorillonite particles.

## Experimental

### Materials

In this study three types of monomers were used for the polymerization process, in addition to the Acrylamide (AA). 3-[Dimethyl-[2-(2 methylprop 2 enoyloxy)ethyl]azaniumyl]propane-1-sulfonate (DMAPS), diallyldimethylammonium chloride (DADMAC) and 2-acrylamido-2 methylpropane sulfonic acid (AMPS) were obtained from Aldrich Chemical Co. Also, ammonium persulfate (APS) was purchased from Sigma-Aldrich company. No further purification of any of the chemicals was performed. The used layered silicates include M^+^ montmorrilonite, Mt, (southern clay mineral products).

### Measurements

The solid copolymer samples were tested using a Galaxy series FT-IR 5000 spectrophotometer at 2 cm^−1^ resolution in the wavenumber range 400–4000 cm^−1^. ^1^H-NMR spectra were recorded on a Bruker Advance 300 MHz instrument by using D_2_O as a solvent. Copolymer thermal stability was assessed using thermogravimetric analysis (TGA) using a Mettler TGA Q5000 TA under nitrogen gas in the temperature range 50–800 °C with a heating rate of 10 °C/min. The weight-average molecular weight (Mw), number-average molecular weight (Mn), and polydispersity index (PDI) of the copolymers were determined using gel permeation chromatography by a K-2301 (KNAUER) detector. The glass transition temperature Tg was recorded on Mettler DSC 2500 TA differential scanning calorimeter at a scanning rate of 10 °C/min under a nitrogen atmosphere. The X-ray diffraction (XRD) of nanocomposites was collected in data using a XPERT-PRO X-Ray diffractometer using Cu Kα radiation (40 kV, 30 mA). The samples were scanned in the range from 2θ = 5° to 15° using step scan of 0.05° for a time interval of 10 s. Also scanning electron microscope (TESCAN, Mira 3-XMU) was used for assigning the surface morphology of the prepared nanocomposites. The contact angle was measured using a digital camera equipped on contact angle tester (JIKAN, CAG-20 SE).

### Polymerization

**Figure 1:**
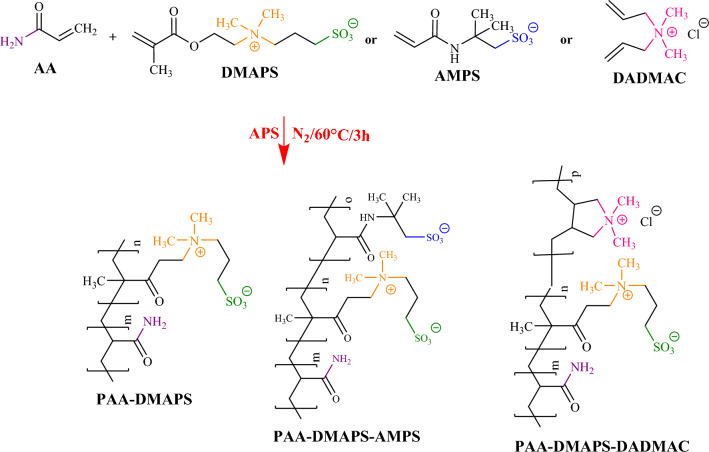
Polymerization of AA copolymers.

AA copolymers were polymerized via conventional free-radical using APS as initiator which were shown in Fig. 1. As a typical procedure, 10 wt% aqueous solutions with various monomer molar ratios in copolymer, according to Table 1, were prepared. The solution was deoxygenated for 15–30 min by nitrogen purge before addition of APS. The polymerization was carried out at 60 °C for 3 h under nitrogen and stirring as long as possible. Only in the presence of anionic monomer (AMPS), it was necessary to adjusted the pH of aqueous solution to around 8.5 before the reaction initiator was added. After the desired time, the synthesized copolymers were separated by precipitation in acetone to remove the remaining monomer, and dried at 40 °C under vacuum for 6 h.

**Table 1:** Monomer reactivity molar ratios in AA copolymers.

Copolymer	AA mol %	DMAPS mol %	AMPS mol %	DADMAC mol %
PAA-DMAPS	75	25	0	0
PAA-DMAPS-AMPS	75	12.5	12.5	0
PAA-DMAPS-DADMAC	75	12.5	0	12.5

### Nanocomposite preparation

A solution of each synthesized copolymers (2 and 5%) in 100 ml graduated cylinder was prepared. Then 0.50 g of montmorillonite was added to all cylinders and shaken for 15 min. The process was photographed periodically to record the formation and sedimentation of the float. After 24 h, nanocomposites were centrifuged for 30 min at 10,000 rpm and sediments were separated.

## Results and discussion

### Fourier transform infrared characterization of copolymers

**Figure 2:**
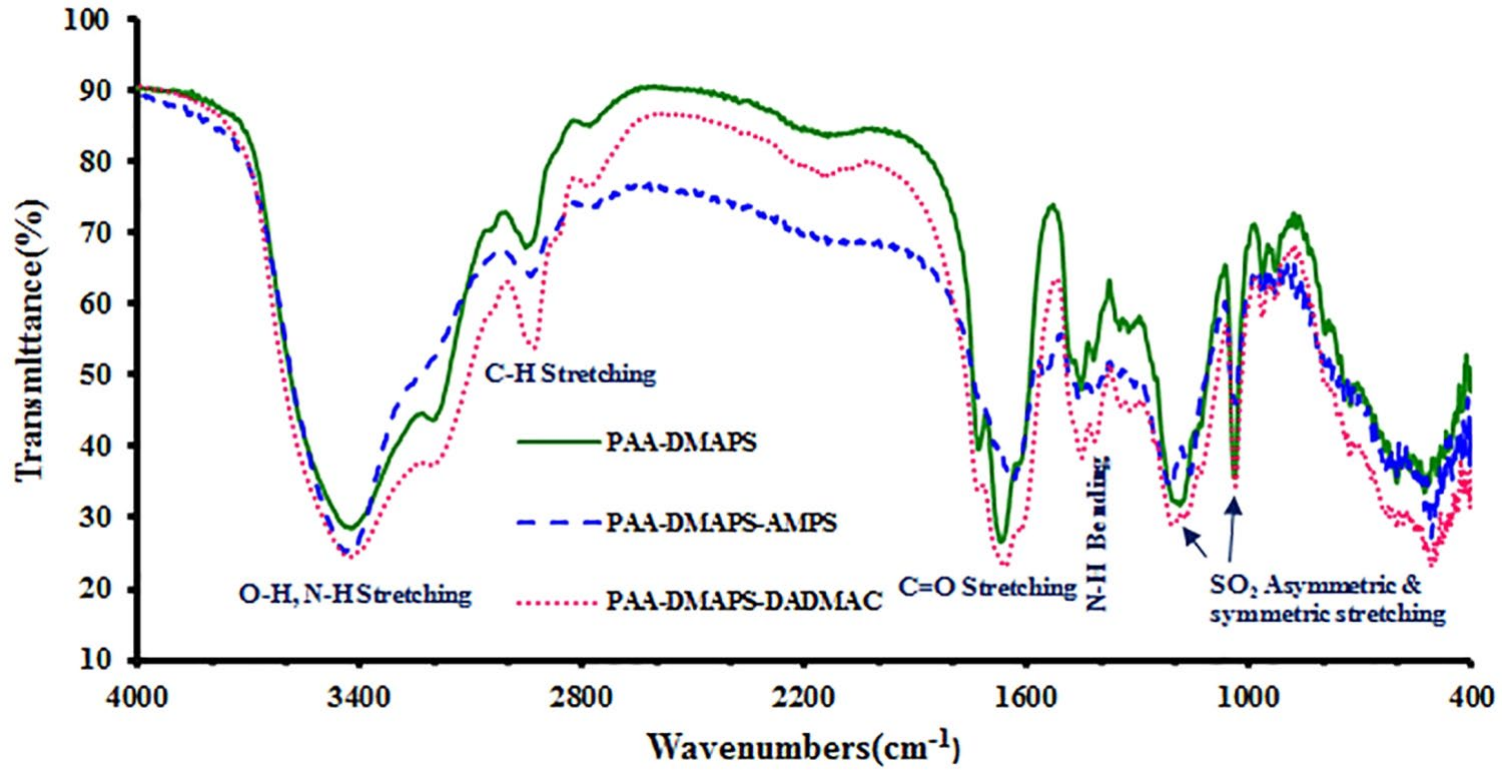
FT-IR spectra of PAA-DMAPS, PAA-DMAPS-AMPS and PAA-DMAPS-DADMAC.

The FT-IR of copolymers are presented in Fig. 2. As the results show, PAA-DMAPS, PAA-DMAPS-AMPS and PAA-DMAPS-DADMAC units exhibit several characteristics bands. The absorption bands at 3420 and 1630 cm^−1^ are due to stretching vibrations of the N–H and C=O bonds. The absorption bands at 1030 and 1160 cm^−1^ assigned to S–O stretching symmetric and asymmetric modes of sulfonic acid groups, respectively. The absorption band at 3375 cm^−1^ is attributed to –OH stretching vibration.

### ^1^H nuclear magnetic resonance (^1^H-NMR) characterization of copolymers

**Figure 3:**
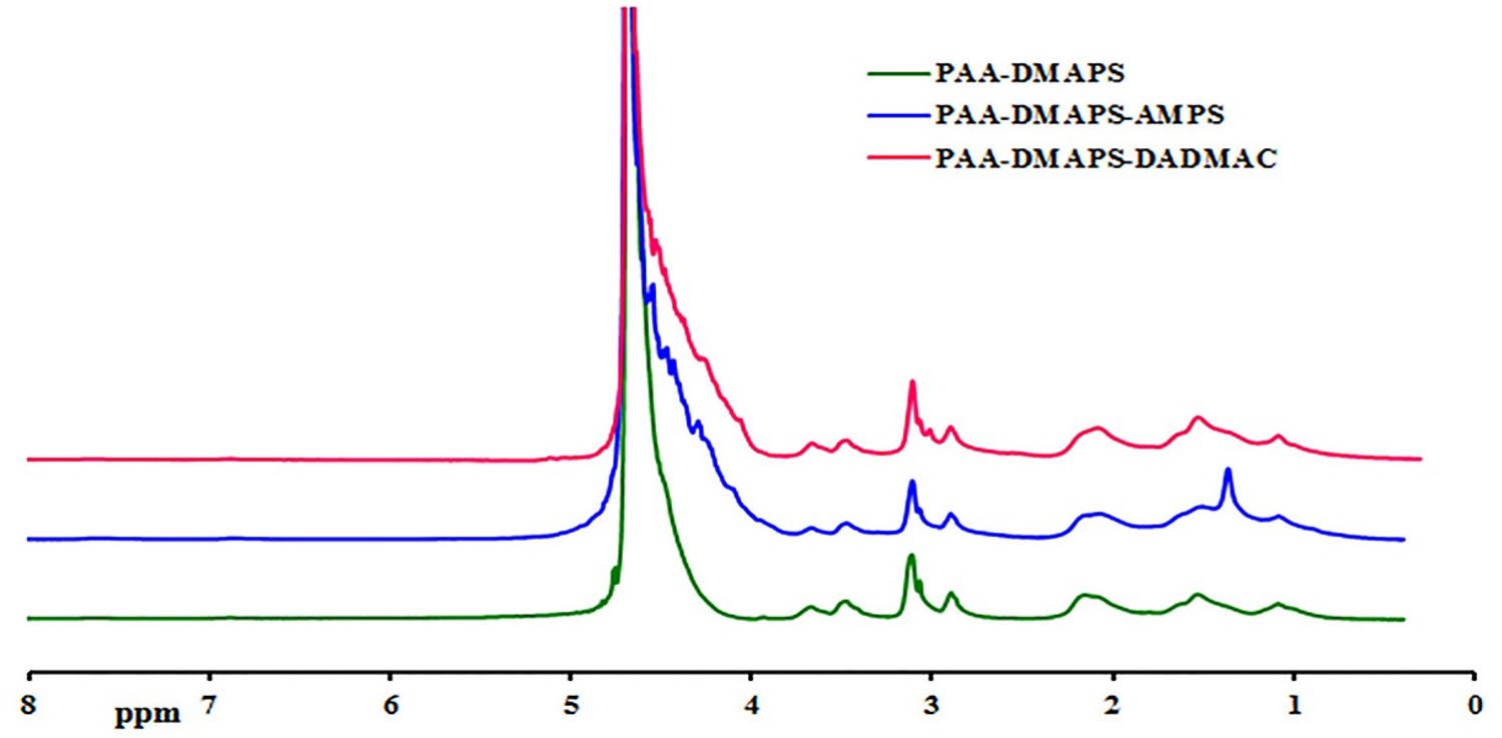
^1^H-NMR of PAA-DMAPS, PAA-DMAPS-AMPS and PAA-DMAPS-DADMAC.

^1^H-NMR analysis was performed to confirm the structure of the copolymers (Fig. 3). All three copolymers have similar ^1^H-NMR spectra with minor differences because they all have AA and DMAPS monomers in their structure. The non-appearance of the resonance of the hydrogens attached to the carbons of the double bond (vinylic hydrogens) is in support of the polymerization reaction.

### Molecular weight measurement

The molecular weight and polydispersity of all prepared copolymers were determined by gel permeation chromatography (GPC). The number average molecular weight (Mn), weight average molecular weight (Mw) and polydispersity index (PDI) of synthesized copolymers are listed in Table 2. The molecular weight of the PAA-DMAPS-AMPS is higher than that of other copolymers due to its greater solubility of AMPS in water. The number-average molecular weight (Mn) and Weight-average molecular weight (Mw) of the PAA-DMAPS-AMPS copolymer (Mn) is about 3,190,000 g/mol and 7,010,000 g/mol, respectively. A good polydispersity index was obtained (PDI = 2.20) for PAA-DMAPS-AMPS.

**Table 2:** Molecular weights and molecular weight distributions of copolymers.

Polymer	Mn (g/mol)	Mw (g/mol)	PDI^a^ (Mw/Mn)
PAA-DMAPS	550,000	2,030,000	3.69
PAA-DMAPS-AMPS	3,190,000	7,010,000	2.20
PAA-DMAPS-DADMAC	1,710,000	4,100,000	2.40

^a^Polydispersity index.

### Thermogravimetric analysis measurement

**Figure 4:**
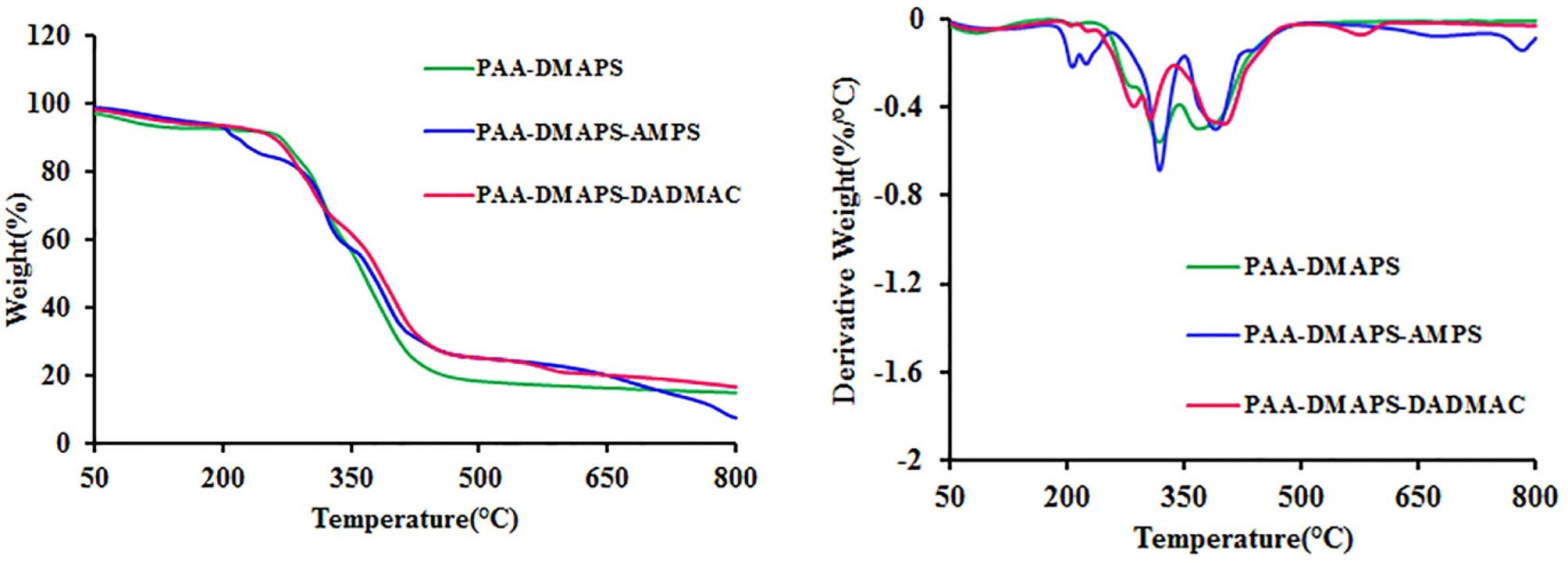
TGA and DTG curves of copolymers.

The curves of thermogravimetric analysis (TGA) of copolymers are given in Fig. 4. The degradation stages and the mass loss, derived from the TGA curves are listed in Table 3. As can be seen, the degradation of all samples proceeds in two stages. The first decomposition stage is assigned to the volatilization of moisture and residual solvent. The onset of the next peak is at approximately 300 °C and represents the starting point of the thermal degradation of copolymers. Decomposition of PAA-DMAPS-DADMAC copolymer occurred at the highest temperature, due to having less N–H groups compared to PAA-DMAPS-AMPS, thus less water removal. On the other hand, it has a lower SO_2_ to remove than the other two samples. The thermal decomposition temperature of PAA-DMAPS is higher than that of PAA-DMAPS-AMPS, because the PAA-DMAPS-AMPS has more N–H groups compared to PAA-DMAPS.

**Table 3:** Thermal characteristic data of copolymers.

Polymer	T_max_ (°C)^a^	Tg (°C)^b^
PAA-DMAPS	342	181
PAA-DMAPS-AMPS	320	170
PAA-DMAPS-DADMAC	405	158

^a^Temperature at the maximum rate of mass loss.

^b^Glass transition temperature.

### Differential scanning calorimetry (DSC) measurement

**Figure 5:**
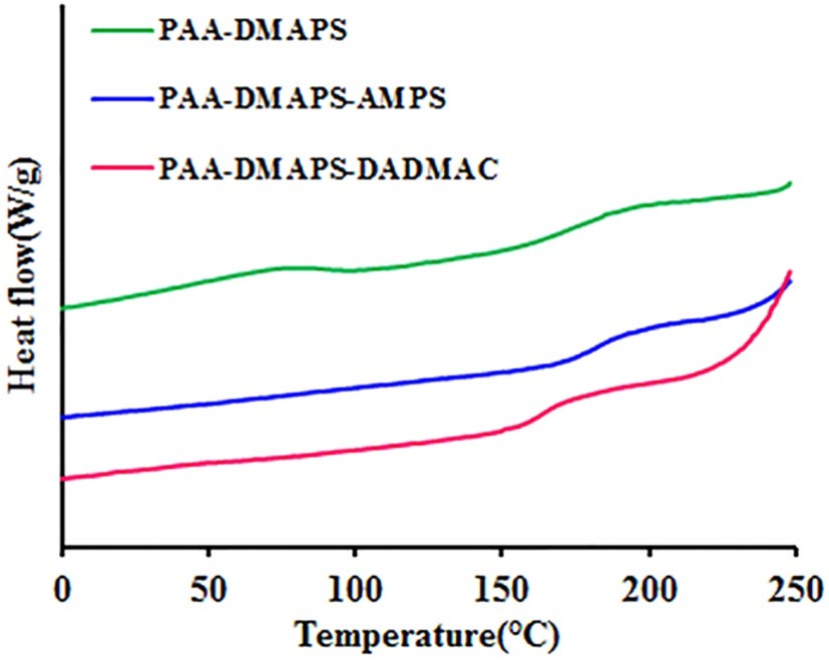
The DSC thermograms of copolymers.

The thermal analysis of copolymers was performed by DSC to obtain the glass transition temperature as an important parameter that determines the mechanical and thermal behavior of the copolymers (Fig. 5). All curves show moisture loss at about 100 °C. As can be seen in these curves, the glass transition temperature for the copolymers are in the range of 158–195 °C, which corresponds to the second order transition (Table 3). The results suggest that the thermal resistance of PAA-DMAPS is higher than that of other copolymers due to its more symmetrical structure and suitable interactions. Also, the glass transition temperature of PAA-DMAPS-AMPS sample is higher than that of PAA-DMAPS-DADMAC copolymer because it has more N–H groups and as a result more number of hydrogen bonds which leads to more interaction of copolymer chains.

### Sedimentation test

**Figure 6:**
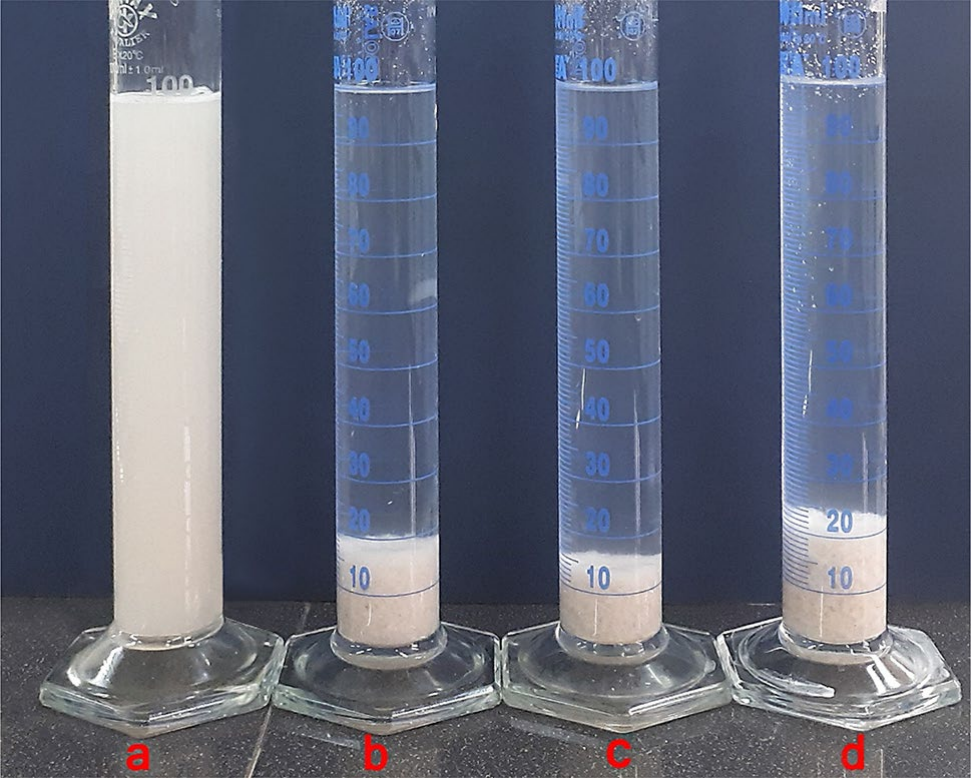
Sedimentation behavior of Mt dispersions in different inhibitor solutions. Left to right: (**a**) Mt; (**b**) Mt + PAA-DMAPS; (**c**) Mt + PAA-DMAPS-AMPS; (**d**) Mt + PAA-DMAPS-DADMAC; (5%, The photo was taken after an hour).

**Figure 7:**
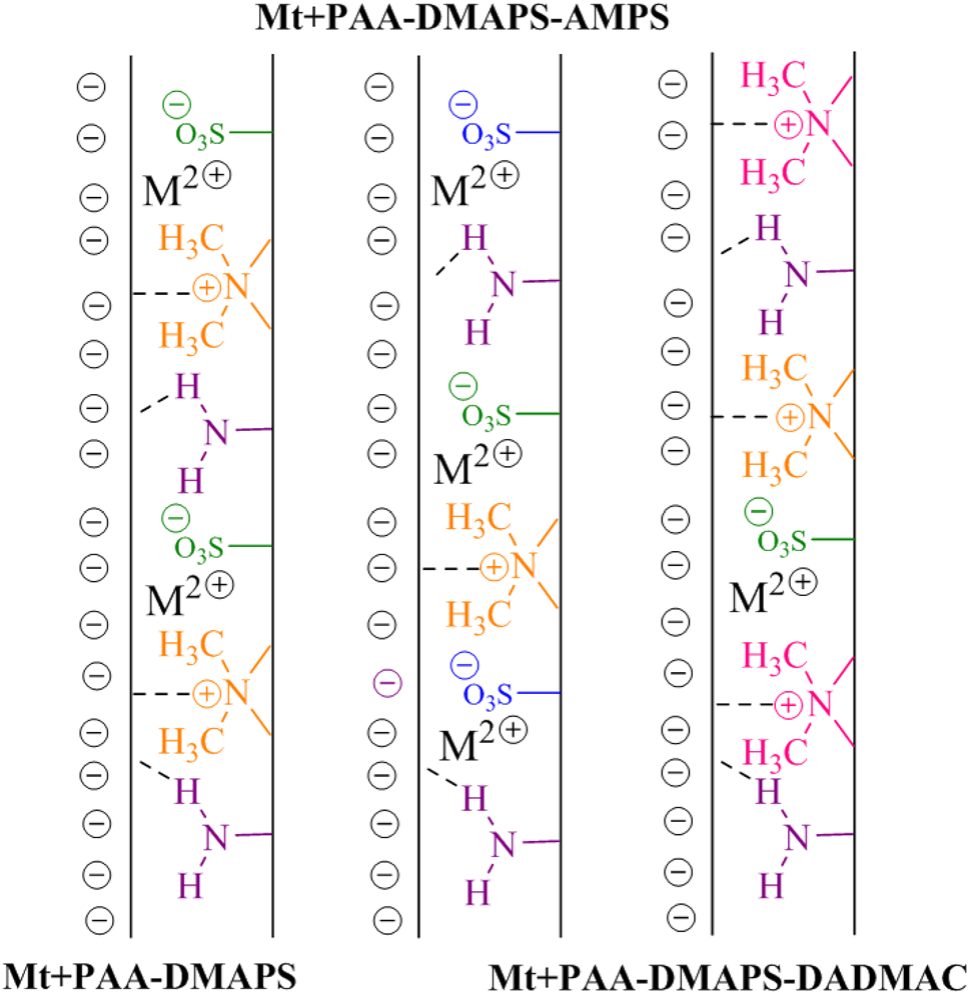
The mechanism of bridging flocculation in different inhibitor solutions.

Figure 6 provides a photographic description of settling behavior of coagulant and flocculant in the set of measuring cylinders. The different electrostatic attraction between opposite charges play an important role in the formation of copolymer clots (Fig. 7)^25^. The electrostatic attraction forces effectively direct the binding of positively charged coagulant to the Mt negatively charged surface of Mt. The PAA-DMAPS-AMPS sample is the best type of flocculant due to its electrostatic attraction and hydrogen bonds. As the anionic groups increase in the PAA-DMAPS-AMPS copolymer, this process causes the adsorption of anions on the cationic metal ions present in the Mt. The PAA-DMAPS-DADMAC coagulant, which has dangling groups with positive charge, has other electrostatic attractions with the Mt surface. The PAA-DMAPS sample has less hydrogen bonds and more electrostatic attractions compared to PAA-DMAPS-AMPS and PAA-DMAPS-DADMAC, respectively. As a result, it is a better coagulant than PAA-DMAPS-DADMAC due to the dominance of ionic interaction effect^12,32,33^.

### Fourier transform infrared characterization of Mt and Mt-copolymer composites

**Figure 8:**
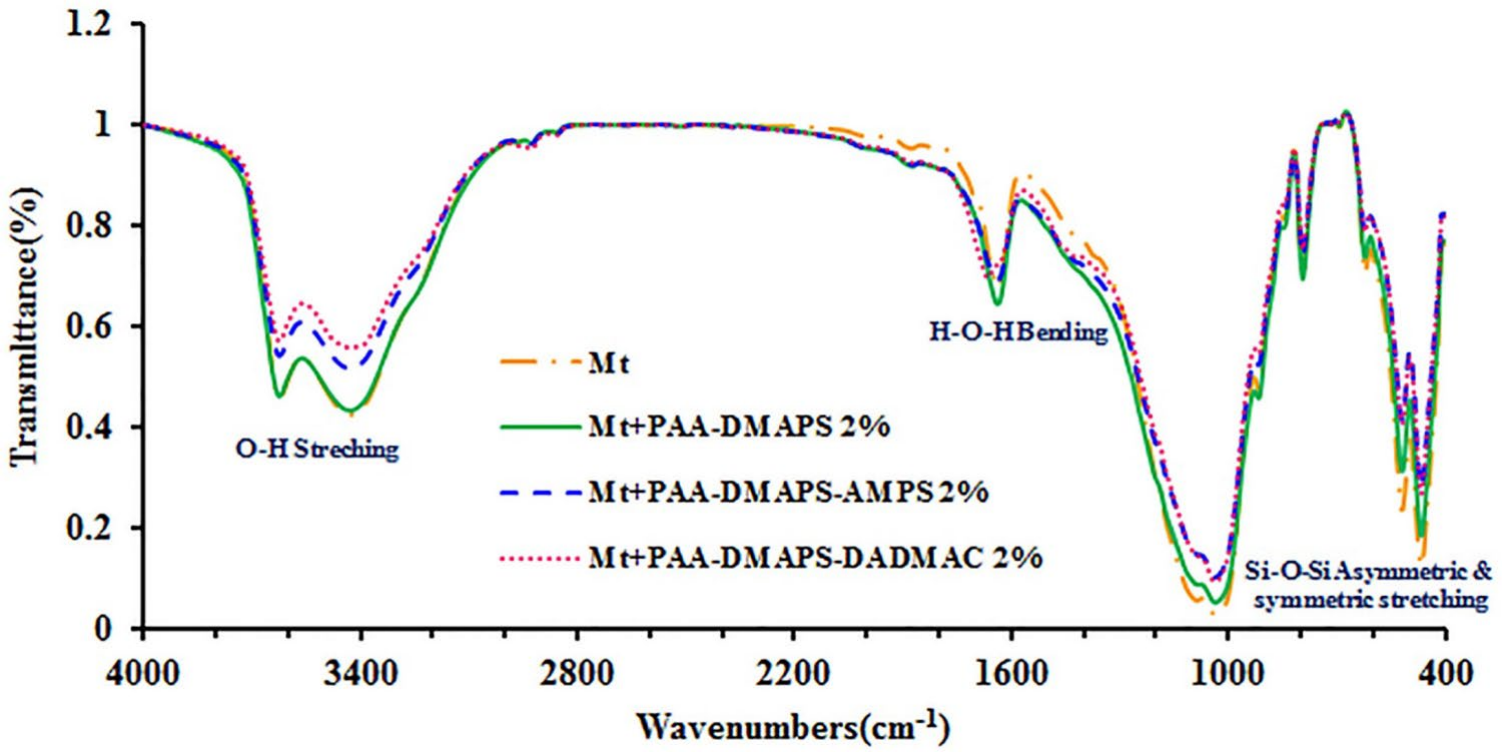
FT-IR spectra of Mt and Mt-copolymer composites.

Figure 8 shows the FT-IR spectra of the Mt and Mt-copolymer composites. The transmittance bands at 1034, 954 and those between 3010–3770 cm^−1^ can be associated, respectively, with Si–O stretching, Si–O bending and –OH stretching^34–36^. Also, another absorption band appeared at about 1636 cm^−1^ which can indicates the presence of the adsorbed water. FT-IR study does not show substantial changes in the structure of Mt-copolymer composites.

### X-ray diffraction (XRD) analysis

**Figure 9:**
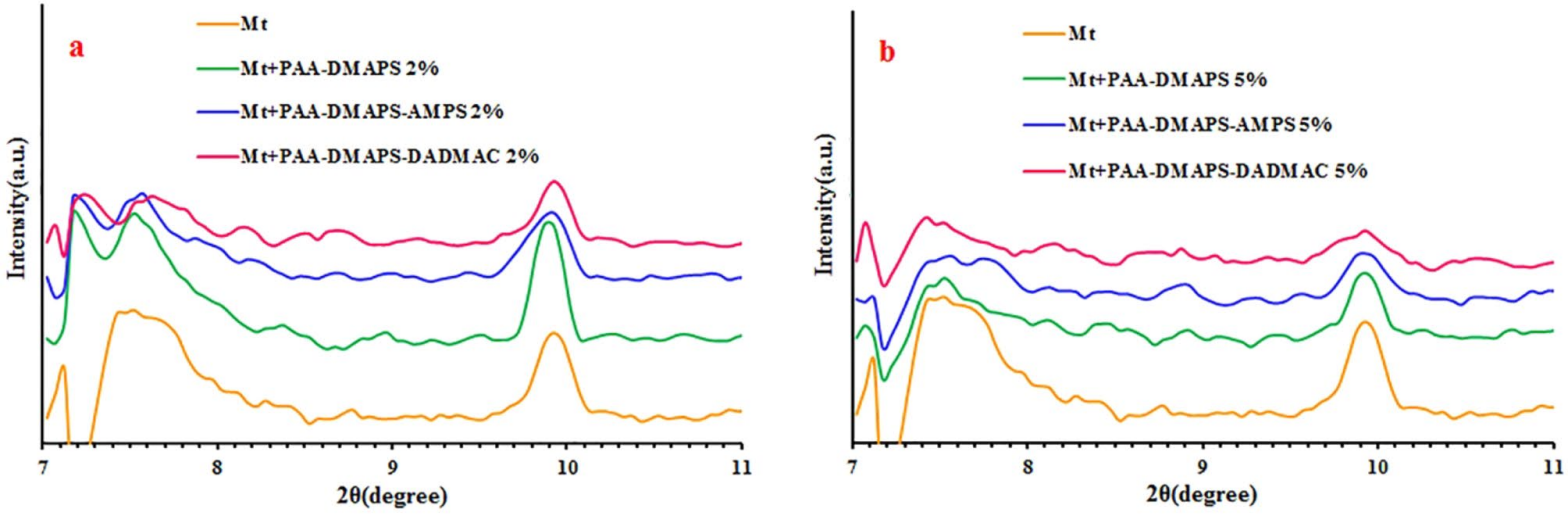
XRD patterns of Mt and Mt-copolymer composites (**a** 2%, **b** 5%).

The d-value can be determined by the diffraction reflection in the XRD patterns, and can be expressed by Bragg’s equation (d = nλ/2sinθ), where d-value is the average distance between Mt layers, θ is the diffraction angle, and λ is inlet X-ray wavelength. The XRD patterns of the Mt and Mt-copolymer (PAA-DMAPS, PAA-DMAPS-AMPS, PAA-DMAPS-DADMAC) composites are showed in Fig. 9. The calculated distance between Mt layers is 1.171 nm. As can be seen from the Fig. 9, the 2θ value of corresponding XRD reflections increase with the addition of copolymers into Mt due to the intercalation of PAA-DMAPS, PAA-DMAPS-AMPS and PAA-DMAPS-DADMAC with the Mt layers (Fig. 9 (a.2%) and (b.5%)) (Table 4). The interlayer space calculated for composites with Mt content of 2% decreases or remain constant. In the Mt + PAA-DMAPS sample, the interlayer space remains constant compared to that of Mt, which indicates the lack of water penetration between the plates. The reason for decreasing the interlayer space in the Mt + PAA-DMAPS-AMPS sample can be attributed to long chain length and better encapsulation of Mt. Also, regarding the Mt + PAA-DMAPS-DADMAC sample, the space between the layers can be reduced due to the increase in the positive charge of the chains and as a result keeping the Mt sheets together.

**Table 4:** X-ray diffraction data of Mt and Mt-copolymer composites.

Polymer	2θ (°)	d-value (nm)
Mt	7.546	1.171
Mt + PAA-DMAPS 2%	7.543	1.171
Mt + PAA-DMAPS-AMPS 2%	7.574	1.166
Mt + PAA-DMAPS-DADMAC 2%	7.629	1.158
Mt + PAA-DMAPS 5%	7.440	1.187
Mt + PAA-DMAPS-AMPS 5%	7.631	1.157
Mt + PAA-DMAPS-DADMAC 5%	7.366	1.199

### Scanning electron microscope observations

**Figure 10:**
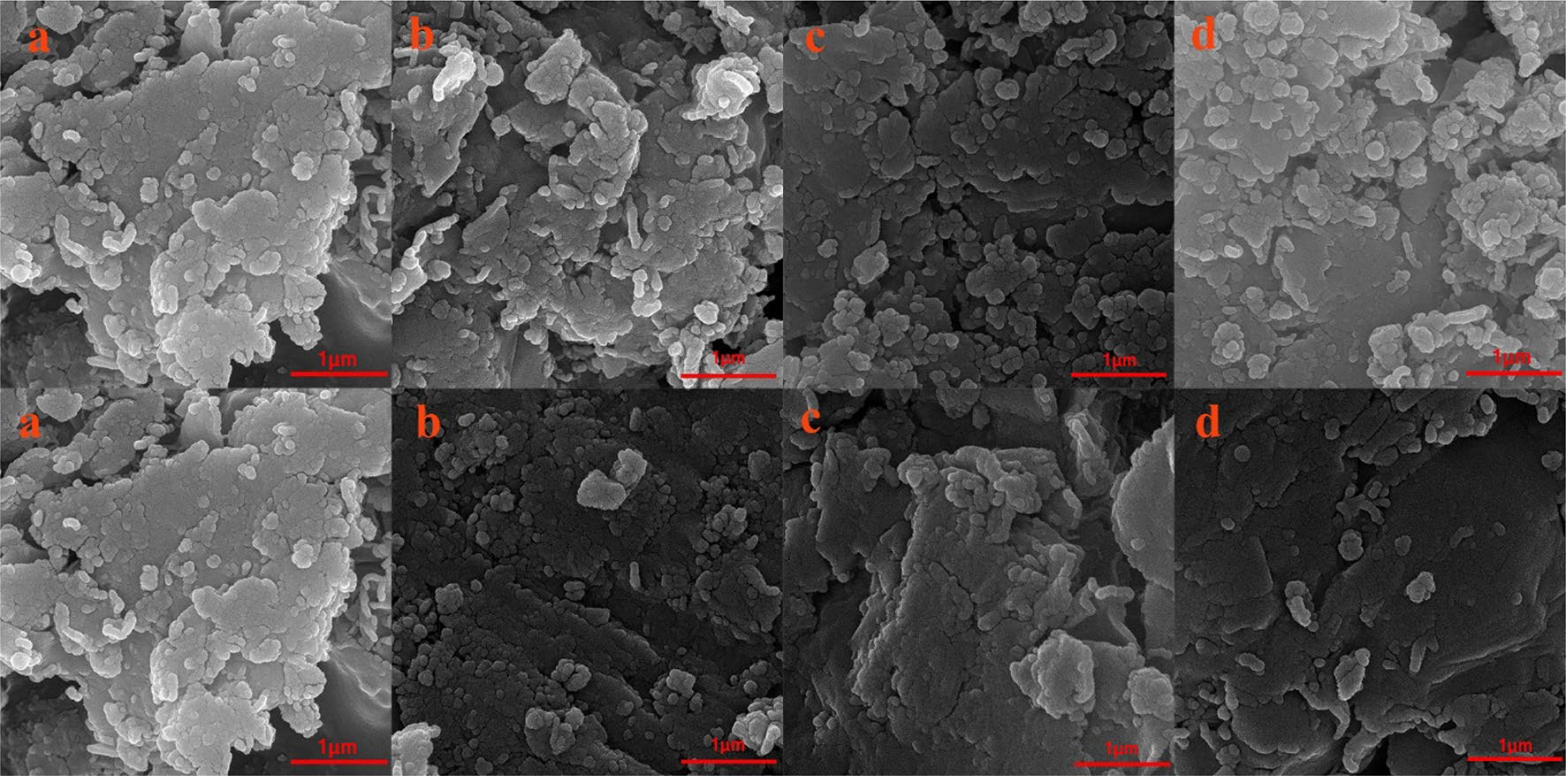
The FE-SEM images of (**a**) Mt; (**b**) Mt + PAA-DMAPS; (**c**) Mt + PAA-DMAPS-AMPS; (**d**) Mt + PAA-DMAPS-DADMAC (Top images are 2% and bottom images are 5%).

Figure 10 presents the SEM images of Mt-copolymer composites and free Mt. The surface of 2% Mt-copolymer composites compared with 5% Mt-copolymer composites exhibits more uniform surface. Mt + PAA-DMAPS and Mt + PAA-DMAPS-AMPS have more uniform dispersion than other copolymers.

### Contact angle measurements

**Figure 11:**
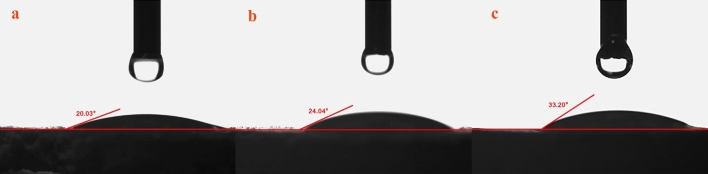
The contact angle measurement of (**a**) Mt; (**b**) Mt + PAA-DMAPS; (**c**) Mt + PAA-DMAPS-AMPS.

Water contact angle measurements (θ) of the Mt and some of Mt-copolymer composites indicated a low degree of surface wetting (Fig. 11a–c). The average value of contact angles determined for all samples describes the surface as hydrophilic. When the small amount of copolymers was incorporated, the water contact angles of nanocomposites significantly increased. The relationship between d-value and contact angle in copolymer composites was investigated. The results showed that when the value of d increases, there is an increase in the contact angles.

## Conclusions

Free-radical copolymerization of the acrylamide copolymers was performed in oxygen-free distilled water by using APS as the initiator at 60 °C. The molecular weight of the synthesized copolymers in different solvents analyzed by GPC. It was found that the acrylamide-amphoter-anion copolymer had the highest molecular weight. Additionally, differences heat resistance between prepared copolymers were monitored via thermogravimetric analysis. All copolymers are thermally stable up to 300 °C. The acrylamide copolymer flocculants are designed based on different charge density. Flocculation properties of copolymers were evaluated by sedimentation volume measurement, XRD, SEM and contact angle measurement. The results showed that the charge density plays an important role in flocculation performance. Flocculation testing proves polyacrylamides are bonded to the surface of montmorillonite particles with electrostatic attraction forces along. The acrylamide-amphoter-anion sample showed the best coagulation efficiency. Most importantly, the acrylamide copolymers demonstrate good biocompatibility, so they are suitable alternatives to other commercial coagulants.

## Data Availability

All data generated or analysed during this study are included in this published article.
